# CO_2_ Laser-Assisted Sclerectomy *vs*. Microcatheter-Assisted Trabeculotomy in the Management of a Bilateral Congenital Ectropion Uveae With Glaucoma: A Case Report and Literature Review

**DOI:** 10.3389/fmed.2022.902716

**Published:** 2022-05-19

**Authors:** Min Chen, Yuhang Li, Bo Cheng, Qi Zhang, Xin Liu, Kaijun Wang

**Affiliations:** ^1^Eye Center, The Second Affiliated Hospital, Medical College of Zhejiang University, Hangzhou, China; ^2^Zhejiang Provincial Key Lab of Ophthalmology, Hangzhou, China; ^3^Department of Ophthalmology, Fenghua People's Hospital, Zhejiang, China

**Keywords:** congenital ectropion uveae, glaucoma, intraocular pressure, CO_2_ laser-assisted sclerectomy surgery, microcatheter-assisted trabeculotomy

## Abstract

**Introduction:**

Bilateral congenital ectropion uveae (CEU) is rare syndrome, usually accompanied by refractory glaucoma. Proper and timely treatment was very important for the prognosis. The report aims to compare the long-term outcomes and complications between the two eyes after different approaches of surgery in a case of bilateral CEU with advanced glaucoma.

**Case Presentation:**

The patient was a 20-year-old male with bilateral CEU and glaucoma. The intraocular pressure (IOP) was 48 mm Hg in the right eye (OD) and 52 mm Hg in the left eye (OS). The vertical cup-to-disc (C/D) ratio was nearly 1.0 in both eyes. Despite maximum medical therapy, the target IOP could not be achieved. Therefore, CO_2_ laser-assisted sclerectomy surgery (CLASS) was performed in OS, and the IOP was remarkably decreased. 1 month after the surgery, the IOP rebounded slightly and was controlled with a fixed-combination anti-glaucoma medicine. 3-month postoperatively, a YAG laser goniopuncture (LGP) was performed to enhance the IOP-lowing effect and the anti-glaucoma agent was discontinued. An ab externo microcatheter-assisted trabeculotomy (MAT) was performed in OD, and the IOP was also significantly decreased. During the follow-up period, the IOP was well controlled for both eyes without any medication. Shallow anterior chamber and complicated cataract developed in OS after CLASS, and there was no obvious late complication in OD after MAT.

**Conclusions:**

To our knowledge, this was the first attempt to perform two different surgeries, CLASS and MAT, in both eyes of a single patient presented with bilateral CEU with glaucoma. Our results showed that the IOP was lower after CLASS, but there were potential complications such as shallow anterior chamber and complicated cataract. MAT could achieve a moderate IOP-lowing effect but had a higher safety. CLASS and MAT may be considered effective surgical options for the management of such patients.

## Introduction

Congenital ectropion uveae (CEU) is an uncommon, non-progressive neural crest cell condition, characterized by the proliferation of iris pigment epithelium on the anterior surface of the iris from the pigment ruff (1). It was first described by Wicherkiewicz (2) and Spiro (3) in the late 19th century. Multiple studies have shown that progressive glaucoma may be present in this entity at birth, infancy, or later in life due to angle dysgenesis (1–5). CEU may be also associated with other ocular abnormalities like ptosis, neurofibromatosis, facial hemihypertrophy, Prader-Willi syndrome, and Rieger's syndrome (4).

The majority of CEU cases were unilateral, only five cases had reported bilateral CEU (4, 5). The main recognized mechanisms of glaucoma associated with CEU involve: 1) developmental angle abnormalities, 2) secondary angle-closure resulting from the iris adherent to the peripheral cornea and trabecular meshwork, 3) iris fibrovascularization, and 4) obstruction of the normal outflow channels caused by the melanin granules released from abnormal iris pigment (6–8). Medication alone was insufficient to achieve the target intraocular pressure (IOP). Most of the patients needed filtering surgery to control the IOP, among whom some underwent multiple operations.

Here we reported a rare case of bilateral CEU with bilateral advanced glaucoma in a 20-year-old male patient, who was managed with new strategies. For the first time, two different approaches, CO_2_ laser-assisted sclerectomy surgery (CLASS) and an ab externo microcatheter-assisted trabeculotomy (MAT) were performed in both eyes of a single patient. The long-term outcomes and complications were compared between OD and OS during a 2-year follow-up period.

## Case Presentation

**Figure 1:**
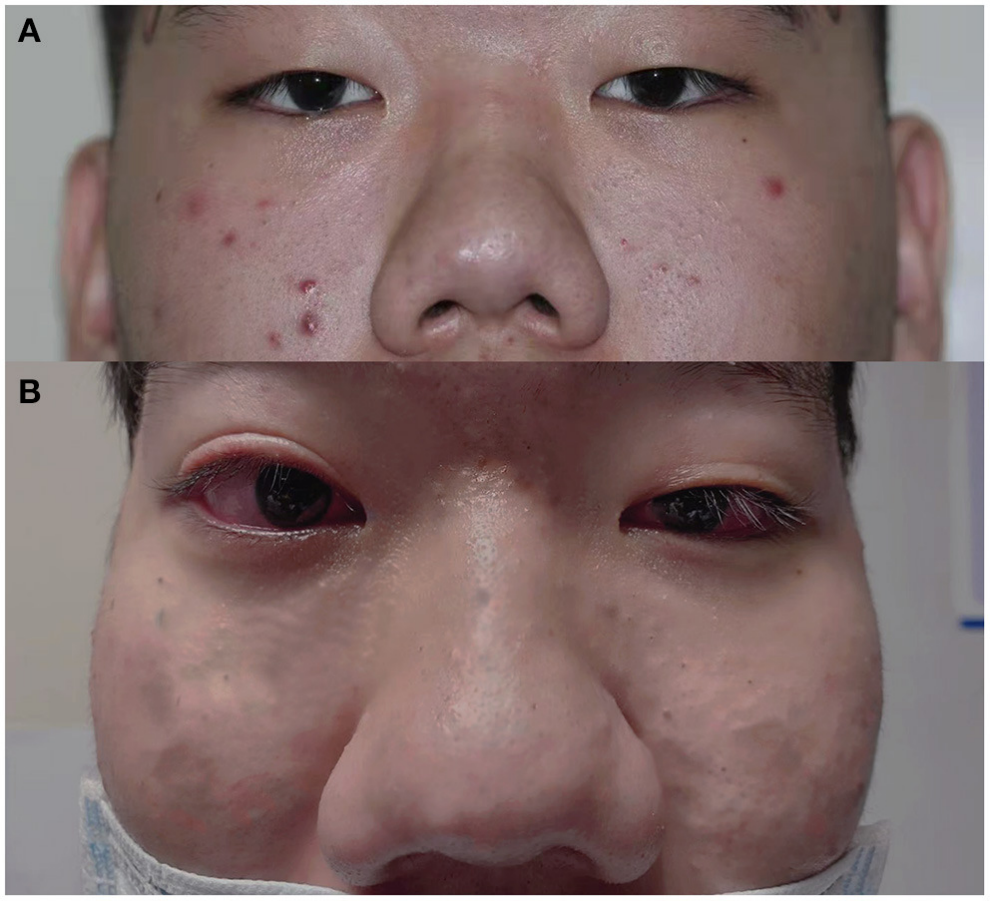
General appearance of the patient. **(A)**: Facial hypertrophy and thickening of the nasal soft tissue can be seen since we first saw him till now. Bilateral mild ptosis before the blepharoptosis correction surgery 3 years ago. **(B)**: Mild ptosis still existed in OS, and incomplete eyelid closure existed in OD after the surgery.

A 20-year-old male came to our eye center in 2019, complaining of progressive painless vision loss in both eyes for the past 3 months. He had bilateral ptosis since birth (Figure 1A) and underwent blepharoptosis correction surgery 3 years ago. He denied any ocular or systemic disease, or any topical or systemic medication history. Family history was negative for congenital ocular abnormalities. IOP condition had never been evaluated in his previous clinical inspections.

**Figure 2:**
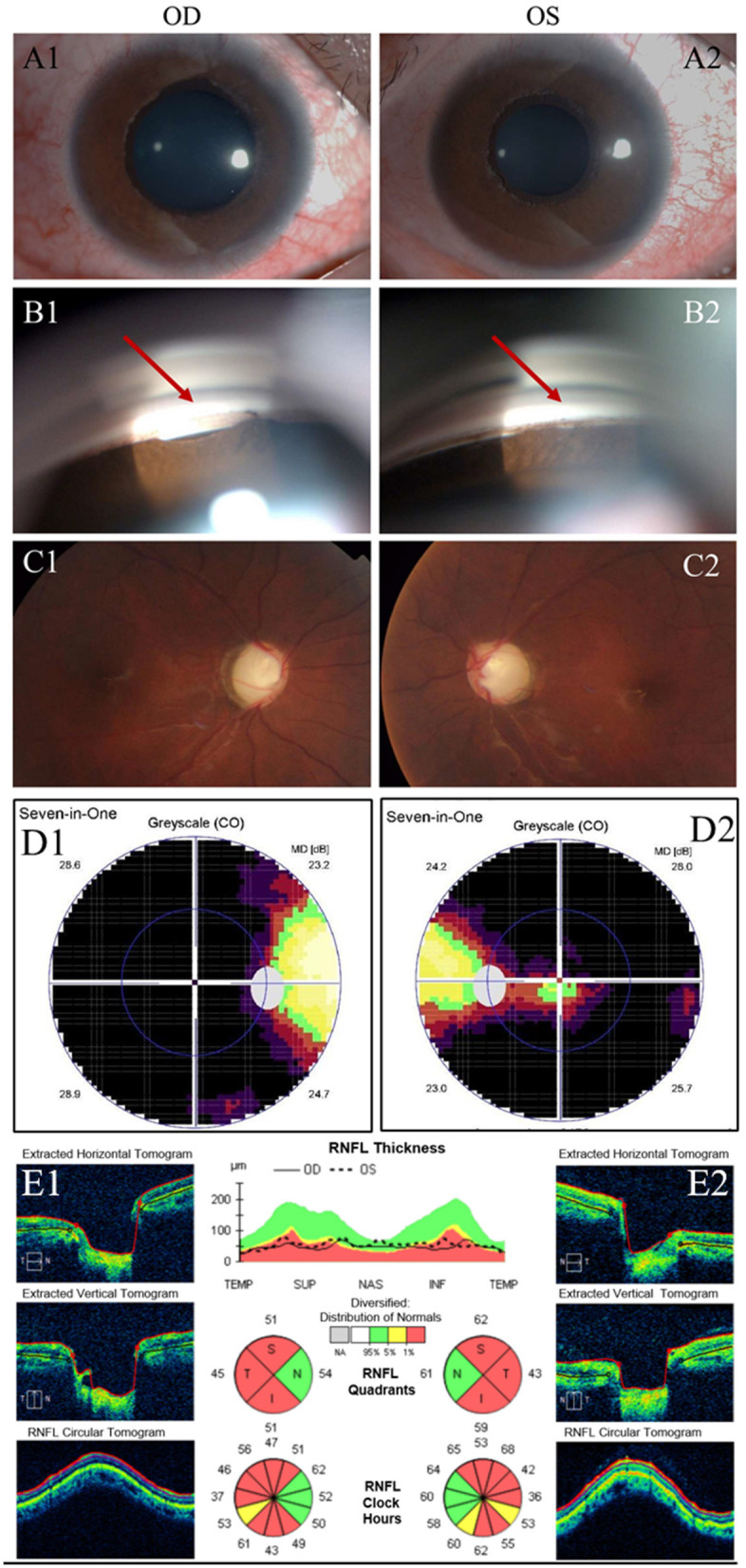
Clinical presentation. **(A)**: Examinations of the patient before surgery. Slit-lamp examination showed 360-degree ectropion uveae in the right **(A1)** and left **(A2)** eyes. **(B)**: Gonioscopy showed anterior insertion of iris in the right **(B1)** and left **(B2)** eyes. (Red arrow) **(C)**: Fundus photos of the right **(C1)** and left eyes **(C2)** showed marked cupping, overpass phenomenon, notching of blood vessels, and thinning of neuroretinal rim at the upper and lower poles. **(D)**: Visual field testing showed temporal islands in right **(D1)** and left eyes **(D2)**. **(E)**: Retinal nerve fiber layer (RNFL) thickness in the right **(E1)** and left eye **(E2)** measured by OCT examination.

Upon examination, the best-corrected visual acuity (BCVA) was 0.3 (logarithm of the minimum angle of resolution, LogMAR) in both eyes, measured by subjective refraction. Myopia was found in both eyes with a spherical equivalent refraction of−6.50/-5.75 diopters without cycloplegia. IOP was 48 mm Hg in OD and 52 mm Hg in OS, measured by Goldmann applanation tonometry (GAT AT900, Haag Streit, Koniz, Switzerland) mounted on a slit–lamp biomicroscope. Mild ptosis still existed in OS, but did not occlude the optic axis. Incomplete eyelid closure existed in OD (Figure 1B). Slit-lamp examination (SL-D8Z; Topcon Corporation, Tokyo, Japan) showed transparent cornea with normal size, clear anterior chamber (AC) with normal depth, and bilateral ectropion uveae in both eyes (Figures 2A1,A2). Gonioscopy revealed abnormal angles with anterior insertion of the iris in both eyes (Figures 2B1,B2). Fundus examination (TRC-NW8; Topcon Corporation, Tokyo, Japan) showed significant pallor of the neuroretinal rim and the vertical C/D ratios of 1.0 in both eyes (Figures 2C1,C2). Central corneal thickness was 519/522 μm, measured by a solid-tip, ultrasonic pachymeter (Tomey SP-3000, Tokyo, Japan). Visual field testing (Octopus 900, Haag-Streit, USA) showed bilateral temporal islands, and <5° central vision in OS (Figures 2D1,D2). The mean retinal nerve fiber layer (RNFL) thickness was 51 μm in OD and 56 μm in OS, measured by optical coherence tomography (OCT) examination (ZEISS CIRROS HD-OCT4000, Germany, Figures 2E1,E2). Systemic physical examination was notable for facial hypertrophy and thickening of the nasal soft tissue (Figure 1). Skin examination was normal, without presentations of café-au-lait spot or any other sign of neurofibromatosis.

Based on the history and examination, he was diagnosed with bilateral CEU with glaucoma, and started on anti-glaucoma eyedrops. During subsequent 3-month follow-up, his IOP was reduced to 29 mm Hg in OD and 27 mm Hg in OS, under maximal glaucoma medications (Ganfort® (Allergan Pharmaceuticals Ireland) once daily; Azopt® (SA Alcon-Couvreur N V) and Alphagan® (Allergan Pharmaceuticals Ireland), three times a day). Due to the severe glaucomatous optic neuropathy, visual field defect and the uncontrolled IOP, prompt surgical intervention was crucial for this patient. Considering the risks and complications of traditional trabeculectomy (Trab) in advanced glaucoma, we performed CLASS (by KJW, Supplementary Video 1) in OS with consent from the patient. The operation went smoothly without intraoperative complications. Postoperatively, the IOP decreased from 27 mm Hg to 7 mm Hg on the first day in OS, and reached 15 mm Hg at 1 week. At 1 month after CLASS, the IOP elevated to 18 mm Hg, which was not low enough for such a young patient with advanced glaucoma. Therefore, a fixed-combination anti-glaucoma medicine (Azarga®, SA Alcon-Couvreur N V) was used twice a day, and the IOP was reduced to 12 mm Hg.

During the follow-ups of OS, the IOP in OD ranged from 20-35 mm Hg despite maximal medications. We decided to choose another surgery for OD, expecting to achieve a lower postoperative IOP without anti-glaucoma medicines. The patient was well-informed and agreed with an ab externo MAT surgery (performed by KJW, Supplementary Video 2) in OD. Hyphema occurred during the surgery, and filled about 1/2 of the anterior chamber on the first day postoperatively. Fortunately, the hemorrhage was spontaneously resolved within 2 weeks, without IOP elevation and any intervention (Supplementary Figure 1). The IOP in OD reduced from 30 mm Hg to 13 mm Hg at 1-week after MAT, and remained stable during the follow-up period.

**Figure 3:**
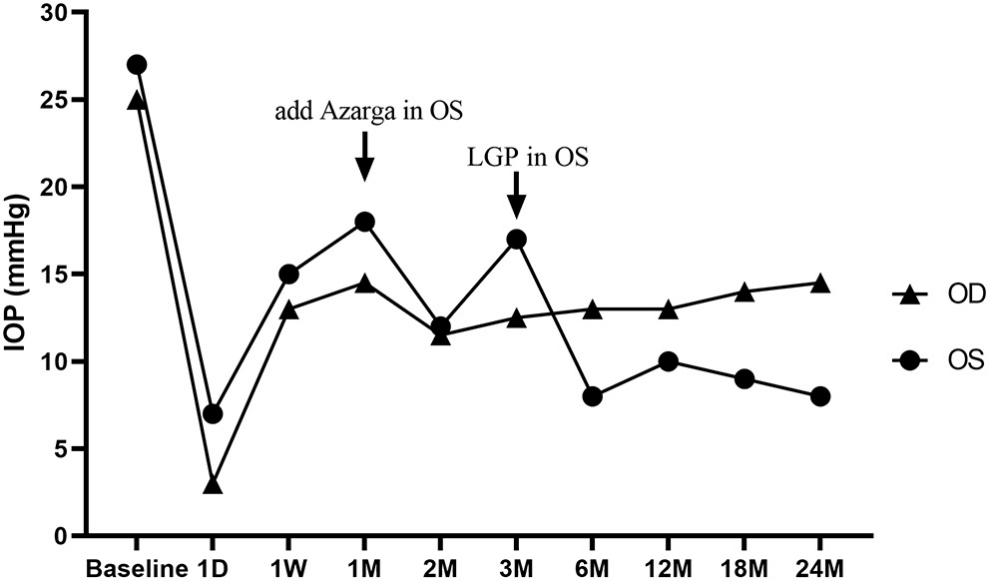
Changes of intraocular pressure (IOP) in both eyes during the 2-year follow-up period. A CO_2_ Laser-assisted Sclerectomy Surgery (CLASS) was first performed in the left eye (OS) and then a Microcatheter-assisted Trabeculotomy (MAT) surgery was performed in the right eye (OD). A fixed combination anti-glaucoma medicine (Azarga®) was added at 1-month post-CLASS, and a YAG laser goniopuncture (LGP) was performed to enhance the IOP-lowing effect at 3-month after CLASS. D: day; W: week; M: month.

Three months after CLASS, IOP in OS was 19 mm Hg under the application of Azarga®. Ultrasonic biomicroscope (UBM) examination indicated scleral lake reduction at the surgical area without peripheral anterior synechia (PAS). Then, a YAG laser goniopuncture (LGP) was performed (by KJW) and the IOP reduced to 6 mm Hg immediately. A diffuse avascular bleb had been maintained in OS since then (Supplementary Figure 2), and the IOP ranged from 8 to 10 mm Hg without any medication (Figure 3). There was no obvious subconjunctival bleb in OD after MAT. No complications occurred in OD during the follow-up period, while a shallow AC and complicated cataract developed in OS (Supplementary Table 1).

**Figure 4:**
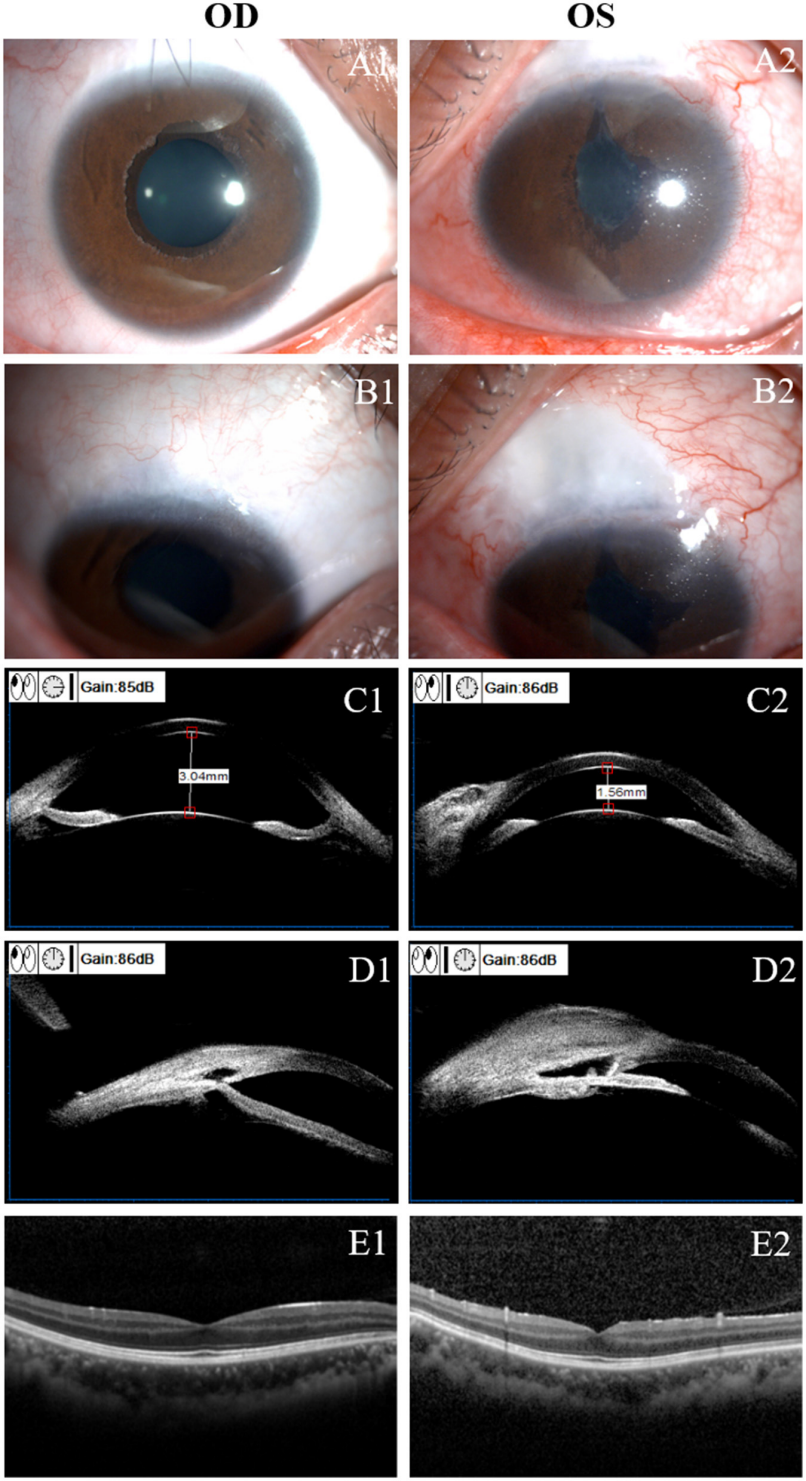
Clinical presentation at 2-year after surgery. **(A)**: Slit-lamp examination of the right (OD, **A1**) and left (OS, **A2**) eye. Shallow anterior chamber and complicated cataract were found in OS. **(B)**: No bleb was found in the right eye **(B1)** and a low-lying diffuse bleb was found in the left eye **(B2)**. **(C)**: Ultrasound biomicroscopy (UBM) examination showed a normal anterior chamber depth in the right eye **(C1)** and a shallow anterior chamber in the left eye **(C2)**. **(D)**: UBM examination showed no obvious bleb in the right eye **(D1)**; a diffuse subconjunctival bleb and a deep scleral lake was found in the left eye **(D2)**. Optical coherence tomography (OCT) examination in OD **(E1)** and OS **(E2)**. There was no obvious hypotony maculopathy in OS.

Due to the impact of the COVID-19 pandemic, the patient failed to come to our hospital for regular follow-ups during the past half-year. He was under close observation at a local hospital, and we kept regular telephone and WeChat follow-up visits with him. At his most recent visit, which was about 2 years postoperatively, the patient's BCVA (LogMAR) was 0.1 in OD and 0.4 in OS. IOP was 14.5 mm Hg in OD and 8 mm Hg in OS with no antiglaucoma agents. There was no obvious hypotony maculopathy in OS by OCT (Heidelberg Engineering, Heidelberg, Germany) examination (Figure 4). During the follow-up period, there was no obvious progression in visual field defect (Supplementary Figure 3). We have recommended antiglaucoma medication to this patient for further IOP reduction in OD, but he refused to use it, with the concern about the incomplete eyelid closure after blepharoptosis correction surgery and potential ocular surface damage caused by local medication. He is currently receiving acupuncture treatment every 2 weeks, which makes him feel better. At present, he is satisfied with the IOP and visual acuity in the right eye. If there is any progression in the VF defect, antiglaucoma medications should be applied. Cataract surgery should be considered in the left eye, if necessary. He is still under close observation.

## Discussion

CEU is defined as the presence of iris pigment epithelium on the anterior surface of the iris (1). Although the mechanisms underlying the development of glaucoma remain complicated, multiple studies have shown the high frequency of refractory glaucoma in CEU (1, 4, 6–31).

So far, there are approximately 100 cases of CEU with glaucoma reported in English literatures. Supplementary Table 2 summarized the clinical features of these patients (1, 4, 6–31). Totally, there were 56 patients (67 eyes), and 61% (*n* = 34) of them were male. The mean age at glaucoma diagnosis was 14.1 ± 9.9 years old and the mean baseline IOP was 38.2 ± 11.4 mm Hg. Ipsilateral mild ptosis was presented in more than one-third of the eyes. If the patient could be diagnosed before blepharoptosis correction and start treatment as soon as possible, the outcome would be completely different for him. CEU may be also associated with other ocular abnormalities such as neurofibromatosis, facial hemihypertrophy, Prader-Willi syndrome, and Rieger's syndrome (32). Therefore, systemic examination should be carefully carried out to detect any remarkable signs of probable associated diseases and avoid misdiagnosis.

Current treatment strategies of CEU with glaucoma include medication, laser treatment and surgery (Supplementary Table 2). Medical management should be attempted initially, but surgery is still the most effective method to control the IOP. Trab with or without antimetabolites is considered a classical and gold standard surgical approach to reduce IOP (33). However, this procedure is associated with a range of potential complications, such as shallow AC, infection, malignant glaucoma, fistula failure, suprachoroidal hemorrhage, etc., which may lead to severe sight-threatening consequences (34).

Unlike the penetrating Trab surgery, CLASS uses a CO_2_ laser to local ablate the scleral tissue, leaving a thin trabeculo-Descemet membrane (TDM) that is just sufficient for aqueous percolation without penetrating the AC, thereby reducing risks of postoperative complications. Zhang et al. (35) compared the effectiveness and safety of CLASS and Trab in a group of Chinese primary open-angle glaucoma (POAG) patients with a 2-year follow-up, which showed a similar IOP-lowing effect but fewer complications and quicker visual recovery in the CLASS group. In our previous study, a IOP reduction of 54.5 ± 16.7% and a total success rate of 95.7% at 1-year after CLASS was reported (36). Although there was anterior insertion of the iris in OS, the whole trabeculum and scleral spur were visible under gonioscopy. Therefore, when surgery was inevitable, we advocated CLASS in OS to avoid severe complications of filtering surgery for this young patient with advanced glaucoma. There was a transient IOP fluctuation during the early stage after CLASS, but reached a satisfactory level after temporary application of anti-glaucoma agent and LGP treatment.

The mechanisms of IOP reduction after CLASS were complicated. There may be several aqueous humor drainage pathways, including subconjunctival bleb, trabecular meshwork, intrascleral outflow, and suprachoroidal outflow (37). It has been reported that the subconjunctival and suprachoroidal pathway may be the main mechanisms to achieve IOP reduction after CLASS (38). For this patient, a diffuse subconjunctival bleb and a deep scleral lack were maintained in OS postoperatively, which contributed to a lower and stable IOP. LGP was an adjunctive procedure after CLASS, which could achieve a further IOP reduction by turning a non-penetrative surgery into a micorpenetrating surgery (39). However, the incidence of shallow AC was relatively low in CLASS than in Trab for POAG patients, with an incidence of 1.5% vs. 6.2% in Caucasian (40) and 0% vs. 21.3% in Chinese patients (35). Complicated cataract has never been reported after CLASS in POAG patients among previous studies (41). In this case, we speculated that due to the maldevelopment of the TM tissue and poor elasticity of the iris, routine LGP treatment with moderate energy resulted in small tears in the thin TDM rather than micro perforation, which promoted overfiltering after CLASS and aggravated the occurrence of shallow AC and complicated cataract. These potential late complications after CLASS deserved further attention, especially in patients with congenital glaucoma.

CEU was a rare type of congenital glaucoma with non-acquired ocular abnormalities. According to the guidelines of World Glaucoma Association: Childhood Glaucoma (Chapter 7), classical initial surgery for primary congenital glaucoma (PCG) is goniotomy or trabeculotomy. 360° trabeculotomy using a catheter to open the whole circumference of Schlemm's canal has been described with favorable results (42). Ab externo MAT has been reported to be an effective and safe treatment for congenital glaucoma (43). Compared with conventional trabeculotomy, MAT showed greater efficacy, with a lower mean IOP, fewer anti-glaucoma medications and maintained a better visual acuity (44). Hyphema was a common post-operative complication and could be absorbed gradually without any intervention in most cases.

For this young patient with bilateral advanced glaucoma, CLASS, an external drainage procedure, was performed in the left eye first, expecting to achieve a target IOP <12 mm Hg. However, he experienced IOP fluctuation during the early stage after CLASS and anti-glaucoma medication was needed. Based on the experience of OS, an internal drainage procedure, MAT, was performed in OD, in order to achieve better outcomes and higher safety for this patient. Although the postoperative IOP was not low enough to reach the target IOP, he gained a better visual acuity postoperatively, without any filtering surgery associated complications. Fontana L et al. reported a IOP reduction from 34.3 ± 9.6 mm Hg at baseline to 14.6 ± 2.3 mm Hg at 2-years after MAT in PCG patients (45), which was consistent with our study.

Comparing the long-term postoperative outcomes between the two eyes, we found that the IOP was lower after CLASS in OS, but there were potential complications such as a shallow AC and complicated cataract. MAT could achieve a moderate IOP-lowing effect but had a higher safety. Doctors often face some grim trade-offs. It was hard to judge which surgery was better than another for this young patient with bilateral advanced glaucoma. The only thing we can do is to try our best. In our opinion, careful decision and individualized treatment according to the specific situation of each patient were helpful to achieve favorable postoperative outcomes.

## Conclusions

In summary, bilateral CEU with glaucoma was not common in clinical practice. We presented not only a rare case but also new treatment strategies for bilateral CEU with glaucoma. For the first time, we performed two different surgeries in the right and left eye of a single patient. Our results indicated that, CLASS and MAT could be considered as effective and alternative surgical options for the management of CEU with glaucoma, especially with advanced glaucoma.

## Data Availability Statement

The original contributions presented in the study are included in the article/Supplementary Material, further inquiries can be directed to the corresponding author.

## Ethics Statement

The studies involving human participants were reviewed and approved by Human Research Ethics Committee, the 2nd Affiliated Hospital, Medical College of Zhejiang University. The patients/participants provided their written informed consent to participate in this study. Written informed consent was obtained from the individual(s) for the publication of any potentially identifiable images or data included in this article.

## Author Contributions

MC and YL worked on drafting the main manuscript. BC worked on preparing figures and collected data from the patients. QZ and XL analyzed and interpreted all data. KW operated the surgery. MC drafted the study and revised it critically for important intellectual content. All authors have read, and approved the final manuscript and publication.

## Funding

This study was supported by National Natural Science Foundation of China (No.82171045).

## Conflict of Interest

The authors declare that the research was conducted in the absence of any commercial or financial relationships that could be construed as a potential conflict of interest.

## Publisher's Note

All claims expressed in this article are solely those of the authors and do not necessarily represent those of their affiliated organizations, or those of the publisher, the editors and the reviewers. Any product that may be evaluated in this article, or claim that may be made by its manufacturer, is not guaranteed or endorsed by the publisher.

## Abbreviations

CEU: congenital ectropion uveae
IOP: intraocular pressure
OD: the right eye
OS: the left eye
CLASS: CO_2_ laser-assisted sclerectomy surgery
LGP: laser goniopuncture
MAT: microcatheter-assisted trabeculotomy
BCVA: best-corrected visual acuity
PAS: peripheral anterior synechia
Trab: Trabeculectomy
AC, anterior chamber. TDM: trabeculo-Descemet membrane.
