# Supplementary material for: CO2 Laser-Assisted Sclerectomy vs. Microcatheter-Assisted Trabeculotomy in the Management of a Bilateral Congenital Ectropion Uveae With Glaucoma: A Case Report and Literature Review
Source: Front Med (Lausanne). 2022 May 19;9:902716. doi: 10.3389/fmed.2022.902716 (PMC9160387; doi:10.3389/fmed.2022.902716)
Supplement: Supplementary file 1 [file Data_Sheet_1.docx]

Supplementary Material

**Supplementary Table 1. Summary of postoperative outcomes in the right and left eyes of the patient.**

| **Date** | **Follow-up time** | | **BCVA (Log MAR)** | | **IOP (mm Hg)** | | **AC depth** | | **Lens** | | **No. of medication*** | | **Treatment** |
| --- | --- | --- | --- | --- | --- | --- | --- | --- | --- | --- | --- | --- | --- |
|  | **OD** | **OS** | **OD** | **OS** | **OD** | **OS** | **OD** | **OS** | **OD** | **OS** | **OD** | **OS** |  |
| 2020/1/10 | - | - | 0.3 | 0.3 | 29.0 | 27.0 | Normal | Normal | Normal | Normal | 4 | 4 | Baseline |
| 2020/1/13 | - | 1D | 0.3 | 0.2 | 35.0 | 7.0 | Normal | Normal | Normal | Normal | 4 | 0 | CLASS in OS |
| 2020/1/22 | - | 1W | 0.5 | 0.2 | 22.5 | 15.0 | Normal | Normal | Normal | Normal | 4 | 0 | - |
| 2020/2/19 | - | 1M | 0.5 | 0.2 | 20.0 | 18.0 | Normal | Normal | Normal | Normal | 4 | 2 | Azarga in OS |
| 2020/3/18 | - | 2M | 0.5 | 0.2 | 25.0 | 12.0 | Normal | Normal | Normal | Normal | 4 | 2 | - |
| 2020/4/16 | 1D | 3M | 0.5 | 0.3 | 3.0 | 17.0 | Normal | Normal | Normal | Normal | 0 | 2 | MAT in OD |
| 2020/4/24 | 1W | 3.5M | 0.5 | 0.4 | 13.0 | 19.0 | Normal | Normal | Normal | Normal | 0 | 0 | LGP in OS |
| 2020/5/8 | 1M | 4M | 0.5 | 0.4 | 14.5 | 7.7 | Normal | Normal | Normal | Normal | 0 | 0 | - |
| 2020/6/19 | 2M | 5M | 0.4 | 0.4 | 11.5 | 7.0 | Normal | Shallow | Normal | Normal | 0 | 0 | - |
| 2020/7/31 | 3M | 6M | 0.4 | 0.3 | 12.5 | 8.0 | Normal | Shallow | Normal | Normal | 0 | 0 | - |
| 2020/8/26 | 4M | 7M | 0.2 | 0.2 | 12.0 | 7.5 | Normal | Shallow | Normal | Normal | 0 | 0 | - |
| 2021/1/29 | 9M | 12M | 0.1 | 0.2 | 14.5 | 10.0 | Normal | Shallow | Normal | Cataract | 0 | 0 | - |
| 2021/7/30 | 15M | 18M | 0.1 | 0.3 | 14.5 | 10.0 | Normal | Shallow | Normal | Cataract | 0 | 0 | - |
| 2021/9/15 | 17M | 20M | 0.1 | 0.3 | 14.0 | 9.0 | Normal | Shallow | Normal | Cataract | 0 | 0 | - |
| 2022/1/16 | 21M | 24M | 0.1 | 0.3 | 15.0 | 7.5 | Normal | Shallow | Normal | Cataract | 0 | 0 | - |
| 2022/3/23 | 23M | 26M | 0.1 | 0.4 | 14.5 | 8.0 | Normal | Shallow | Normal | Cataract | 0 | 0 | acupuncture |

OD: Right eye; OS: Left eye; BCVA: Best-corrected visual acuity; IOP: Intraocular pressure; AC: Anterior chamber; CLASS: CO_2_ laser-assisted sclerectomy surgery; MAT: Microcatheter-assisted trabeculotomy; LGP: Laser goniopuncture; D: Day; W: Week; M: Month; *Fixed combination medications were recorded as two antiglaucoma agents.


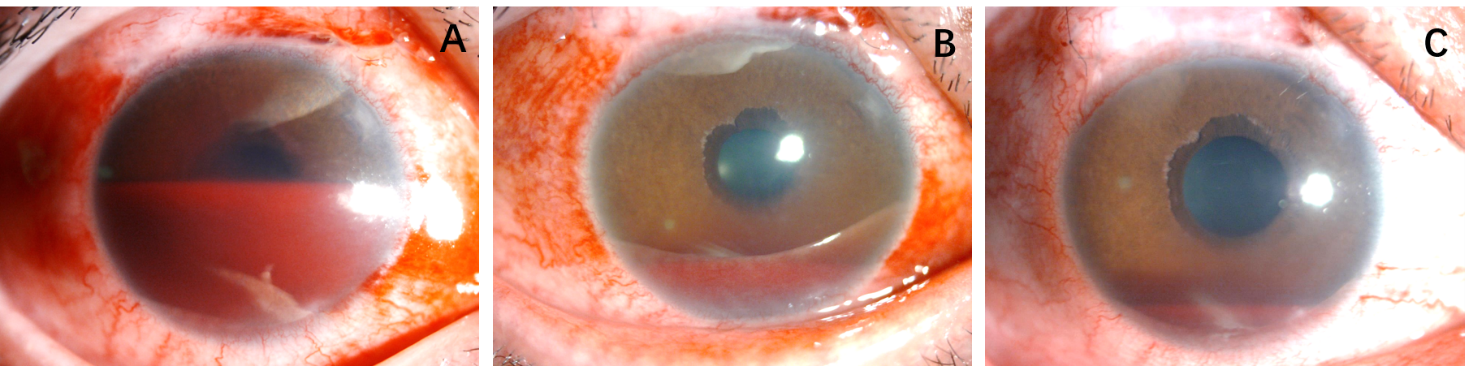


**Supplementary Figure 1. Hyphema in the right eye after MAT.** A: Hyphema filled about 1/2 of the anterior chamber on the first day postoperatively with a IOP of 3 mm Hg. B: Hyphema gradually absorbed at 3 days postoperatively with a IOP of 5 mm Hg. C: Hyphema decreased at 1 week postoperatively with a IOP of 8 mm Hg.


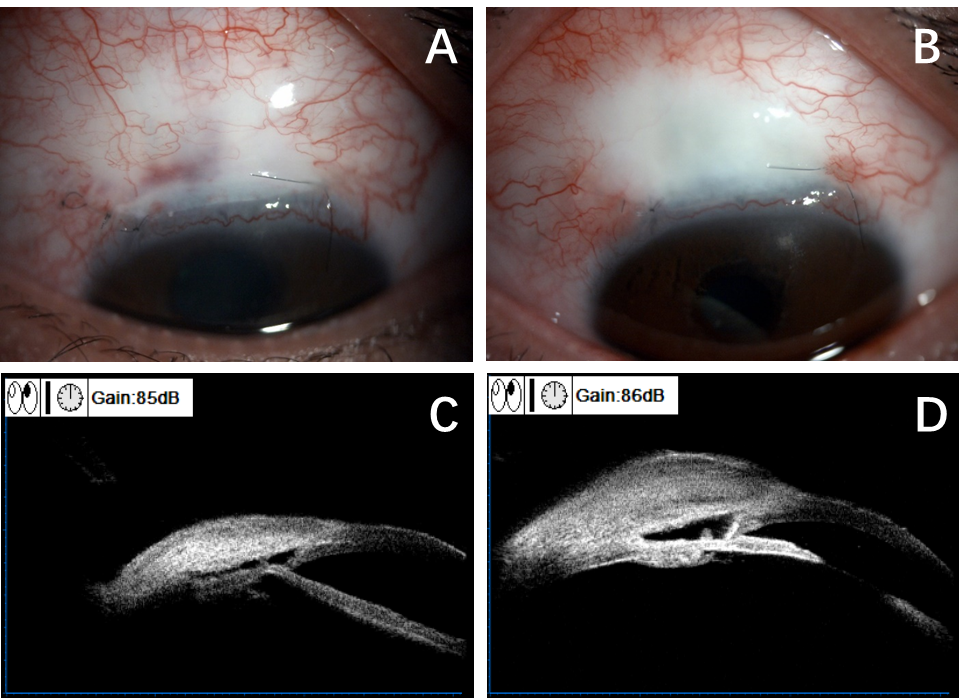


**Supplementary Figure 2. Changes of subconjunctival bleb and scleral lake after LGP treatment in the left eye.** A: Slit-lamp photograph of subconjunctival bleb before LGP. B: Slit-lamp photograph of subconjunctival bleb after LGP. C: Ultrasonic biomicroscope (UBM) examination indicated scleral lake reduction at the surgical area. D: UBM examination after LGP treatment showed a diffuse bleb and a deep scleral lake.


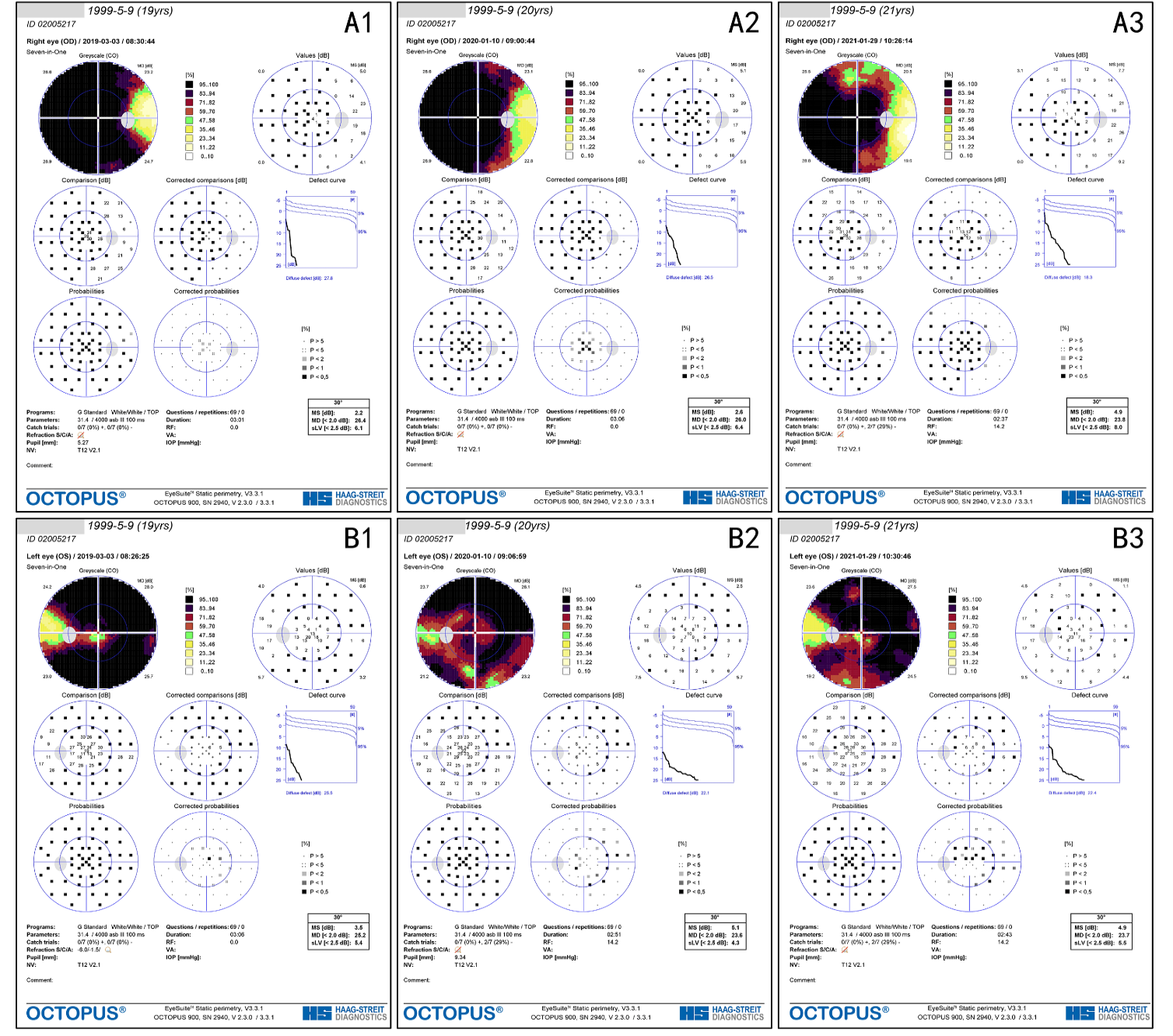
**Supplementary Figure 3. Changes of visual field (VF) during the follow-up period.** A1: VF in OD at the initial visit in 2019. A2: VF in OD at baseline before MAT in 2020. A3: VF in OD at 9 months after MAT in 2021. B1: VF in OS at the initial visit in 2019. B2: VF in OS at baseline before CLASS in 2020. B3: VF in OS at 1 year after CLASS in 2021.
